# Generating Edge Cases for Testing Autonomous Vehicles Using Real-World Data

**DOI:** 10.3390/s24010108

**Published:** 2023-12-25

**Authors:** Dhanoop Karunakaran, Julie Stephany Berrio Perez, Stewart Worrall

**Affiliations:** 1 Emerging Technologies, IAG, Sydney, NSW 2000, Australia; dhanoop.karunakaran@iag.com.au; 2 Australian Centre for Robotics, University of Sydney, Sydney, NSW 2008, Australia; stephany.berrioperez@sydney.edu.au

**Keywords:** autonomous vehicles, testing, edge case generation, scenario-based testing, parametric representation, data-driven method

## Abstract

In the past decade, automotive companies have invested significantly in autonomous vehicles (AV), but achieving widespread deployment remains a challenge in part due to the complexities of safety evaluation. Traditional distance-based testing has been shown to be expensive and time-consuming. To address this, experts have proposed scenario-based testing (SBT), which simulates detailed real-world driving scenarios to assess vehicle responses efficiently. This paper introduces a method that builds a parametric representation of a driving scenario using collected driving data. By adopting a data-driven approach, we are then able to generate realistic, concrete scenarios that correspond to high-risk situations. A reinforcement learning technique is used to identify the combination of parameter values that result in the failure of a system under test (SUT). The proposed method generates novel, simulated high-risk scenarios, thereby offering a meaningful and focused assessment of AV systems.

## 1. Introduction

The Society of Automotive Engineers (SAE) outlines six levels of driving automation [1]. As automation levels increase, autonomous systems must ensure safety without human intervention. Therefore, comprehensive evaluations are crucial before deploying these systems on public roads. Unlike deterministic approaches like distance-based testing, scenario-based testing (SBT) offers a promising alternative by assessing systems against meaningful driving scenarios and reducing test efforts for safety assurance [2,3,4]. SBT concentrates on creating high-risk traffic situation test cases for evaluating a system’s performance. In SBT, there are three levels of representations [5]:

- *Functional scenarios,* which comprise the highest abstraction layer and contain a linguistic description of a scenario.
- *Logical scenarios,* which detail the range and distribution of parameters that describe a specific event.
- *Concrete scenarios,* which arise from specifying a value for each parameter, and are sampled from the distribution defined in the logical scenario.

Moreover, concrete scenarios can be classified into the following three types based on their occurrence probability: *typical*, *critical*, and *edge case* scenarios.

- *Typical scenarios* represent common, real-world operating behaviors with a low likelihood of leading to high-risk situations.
- *Critical scenarios* entail higher-risk situations with safety concerns, occurring less frequently than *typical scenarios*.
- *Edge cases* refer to statistical outliers, presenting challenging scenarios that are rarely encountered in normal driving activities.

Identifying a combination of parameter values that describe realistic scenarios is challenging due to the complex, non-linear relationships between parameters. A considerable effort is required to pinpoint the optimal combination of parameters that accurately capture all aspects of a given scenario.

Highly automated vehicles (HAVs) will eliminate the need for human drivers, making it crucial to evaluate their operation in edge case scenarios to ensure safe deployment [6]. A representative test case, for example, is a cut-in scenario where the HAV might collide during a lane change.

In the literature, generating concrete scenarios for critical events is a burgeoning research area in SBT. Prior studies have centered on manually constructing simulated scenarios utilizing expert knowledge [7,8,9]. However, these methods may produce scenarios that do not accurately reflect real-world traffic participant behavior. Moreover, many existing techniques focus on scenario generation without giving adequate attention to edge cases [2,10,11]. A recent study by De Gelder et al. applied kernel density estimation to fit a distribution to three parameters from a given scenario [2]. This work generates test cases using Monte Carlo simulation but does not explicitly direct the search toward edge cases.

Generating high-risk scenarios for testing HAVs is important in ensuring their safety and reliability. These scenarios, particularly edge cases, are crucial for assessing how these vehicles perform in rare or unexpected situations, thereby establishing public trust and meeting regulatory safety standards. By simulating realistic driving conditions, including rare and unusual situations, these tests provide a comprehensive evaluation of autonomous systems, thus uncovering potential weaknesses that might not be evident in normal driving conditions. This approach is more efficient than traditional distance-based testing methods and crucial for the overall advancement of autonomous vehicle technology. It allows for focused and effective testing, thus ensuring the vehicles are well equipped to handle a wide range of driving scenarios.

This paper presents a method for generating realistic and challenging concrete scenarios to evaluate HAV subsystems using real-world data. We exemplify our approach using lane change events on urban roads. However, the proposed methodology can be extended to accommodate various road scenarios, thereby highlighting its versatility and applicability. In this work, we used the CARLA simulator, a tool that is engineered to facilitate autonomous urban driving system development, training, and validation [12]. This versatile software allows us to control all digital assets, which encompasses static and dynamic actors, thus enabling the creation or playback of diverse scenarios.

We transformed raw data into meaningful scenarios through a three-step process. First, we extracted lane change maneuvers from the collected data and converted them into a parameterized representation [13], thereby validating the parameterization’s effectiveness using metrics to compare the parameterized scenario representation with the original real-world trajectory. Next, we constructed a search space, or *parameter space*, using extracted parameter values, and we then compared the following three different representations: independent univariate normal, multivariate normal, and a multimodal–multivariate distribution. Finally, we treated CARLA’s open-source simulation tool’s collision avoidance system (CAS) as the system under testing (SUT). We employed an RL method to optimize the parameter values for realistic and challenging scenarios, with the reward function biasing learning toward edge cases.

We summarize our paper’s contributions as follows:

- A novel method for identifying and parameterizing real-world lane change scenarios, thereby demonstrating a strong resemblance between reproduced and real-world trajectories.
- A unique urban lane change dataset (81 cut-in and 53 cut-out) with raw parameter values and OpenSCENARIO representations for each event (available online).
- An extended data-driven methodology [14] for generating problematic concrete scenarios for a specific CAS, which was evaluated through quantitative and qualitative analysis.

## 2. Related Work

This section provides an overview of the previous works on scenario extraction methods, scenario datasets, and concrete scenario generation methods.

De Gelder et al. in [15] implemented a scenario extraction method that relies heavily on real-time tagging. Although the technique enabled automated tagging in real-time and used a combination of tags to mine scenarios, extracting scenarios using other published datasets is not easy. Moreover, the approach was highly dependent on the accuracy and consistency of the real-time tagging process, which may not be reliable. In contrast, Krajewski et al. in [16] demonstrated a more robust scenario extraction process for lane change maneuvers by measuring when the vehicles crossed a lane in the data collected from cameras fitted to drones. This method did not depend on real-time tagging, which made it more reliable and easier to apply to other datasets. Xinxin et al. in [17] presented a scenario extraction framework that relied on various computer vision techniques to extract scenarios from video data. While this approach showed promise, it also had limitations, particularly regarding the accuracy of the extracted scenarios. Similarly, the study of  [18] proposed a methodology to generate concrete scenarios by extracting scenario parameters from the HighD dataset for assessing an active lane-keeping system (ALKS). While their approach was promising it also had limitations, particularly regarding the complexity of the generated scenarios and the need for accurate data to ensure their validity. Despite their limitations, both of the studies of  [17,18] proposed scenario extraction frameworks and generated scenarios in a standard format, which may be helpful for researchers and practitioners in the field.

Several literature contributions have focused on the publication of verification and validation datasets. For instance, the Safety Pilot Model Deployment (SPMD) [19] program, launched by the University of Michigan Transportation Research Institute (UMTRI) with the support of various US departments, collected 73 miles of data and stored it in a text format. However, extracting scenarios from this dataset requires extensive post-processing, which can be time-consuming and inconvenient for end users [20]. Krajewski et al. [16] published the HighD dataset, one of the most extensive highway vehicle trajectory datasets. While it consists of over 16 h of measurements from six locations with around 100,000 vehicles, the trajectories were recorded from a bird’s eye perspective. In the study of [18], a method to extract scenario parameters from the HighD dataset to create concrete scenarios was proposed. While this method is promising, it focuses primarily on highway scenarios, which limits its usefulness for researchers and practitioners interested in urban driving scenarios.

The generation of concrete scenarios has been explored in the literature, but these methods have limitations that must be considered. For instance, Barbier et al. proposed in [10] a technique to evaluate a system’s behavior by formulating it as a statistical model-checking problem. While they computed the statistical characteristics of the system under test (SUT) by identifying the system failures in randomly generated scenarios in a simulation, they did not bias their search toward edge cases. In contrast, in [2], real-world scenarios were parameterized and stored in a database. Monte Carlo simulations were then employed to generate test cases from the parameterized representation. However, the Monte Carlo simulation is a random search. Depending on the scenario, it may be less likely to find a falsification result than a directed search, as described in this paper. Zhao et al. in [11] extracted a statistical model of vehicle behavior from real-world data in a lane following scenario, and they then skewed the statistical model to create more critical scenarios. The performance of this model-based approach depends on having an adequate model representation of real-world behavior, which may not always be the case.

Recent research has focused on generating concrete scenarios using a manually built combination of parameters. For instance, Gangopadhyay et al. proposed using Bayesian optimization (BO) to create challenging scenarios [7]. BO utilizes the Bayes rule to learn the model, and it then finds challenging scenarios by using the learned model. Similarly, in [8], an evolutionary algorithm (EA) was employed to search for critical scenarios from parameter space. Also, Zhou et al. [21] introduced a challenging scenario generation approach for automated driving systems through using genetic-based algorithms. Other researchers, such as Koren et al., Liu et al., and Lu et al. [9,22,23], have employed an RL-based approach to search for collision scenarios from a manually built search space. These methods do not account for real-world traffic participant behavior in the scenario generation process, as their parameter space does not consider the correlations between the different variables.

The authors in [24] presented a novel scenario generation method that utilizes kernel density estimation (KDE) to approximate the probability density function (PDF) of scenario parameters, thus enabling the generation of realistic scenarios. In addition to that, they introduced a novel metric to quantify the extent to which the generated scenarios reflect real-world scenarios. This work was limited as it did not explicitly demonstrate the generation of more critical scenarios.

In our previous work, ref. [14], we proposed a method to generate concrete scenarios for assessing the performance of CAS at pedestrian crossings. Our approach had the advantage of biasing the learning toward high-risk events, which enabled the creation of many concrete scenarios through using a parameter space built from expert knowledge. However, it did not fully encode realistic traffic participant behavior, as expected in a data-driven approach. In this paper, we address this limitation by building the parameter space from real-world data, which enables the generation of more realistic and challenging scenarios in the context of a lane change maneuver.

## 3. Background

This section introduces several fundamental concepts used in our approach to generate concrete scenarios for assessing the performance of collision avoidance systems. These concepts include REINFORCE RL, responsibility sensitive safety (RSS), OpenX formats, the data collection vehicle, coordinate frames, and the lane change scenario types used in our method.

- Reinforce RL

Reinforcement learning (RL) algorithms aim to find an optimal policy that maximizes reward by interacting with the environment modeled as a Markov decision process (MDP) [25]. RL is typically implemented in three ways: dynamic programming, Monte Carlo methods, and temporal difference learning. Our approach employs the Monte Carlo method REINFORCE, a policy gradient algorithm that directly manipulates the policy to find the optimal one that maximizes expected return [26]. In this algorithm, the policy is defined by the weights of the neural networks [27]. The learning process updates the weights to find the optimal policy that predicts the desired action given a state.

- Responsibility Sensitive Safety (RSS)

Responsibility Sensitive Safety (RSS) is a formal method proposed by Intel’s Mobileye that computes the minimum distance required to keep a vehicle safe [28]. RSS aims to guarantee that an agent will not cause an accident rather than to ensure that an agent will not be involved in an accident [28]. Our work focuses on the safe longitudinal distance, as well as on the minimum distance required for the ego vehicle to stop in time if a vehicle or object in front brakes abruptly.

- OpenX Formats: OpenSCENARIO and OpenDRIVE

The OpenX formats, including OpenSCENARIO and OpenDRIVE, enable the construction of simulations based on real-world scenarios using programs that support the format [29,30,31]. These formats facilitate the sharing of test scenarios that have the potential to influence safety profoundly. OpenSCENARIO describes the dynamic contents, such as the behavior of the traffic participants and weather conditions, while OpenDRIVE can represent a road network and the surrounding environment. Our approach uses version 1.1 for both formats, stored as XML files.

- Data Collection Vehicle

Our data collection vehicle is a Volkswagen Passat station wagon fitted with Ibeo and SICK lidars. The Ibeo HAD feature fusion detection and tracking system provides tracking data for capturing the trajectory of dynamic traffic participants. The algorithms and methodology presented in this paper are not specific to this exact sensor arrangement. It is possible to employ this framework on a different platform with minor modifications to the code.

- Coordinate Frames

Our scenario extraction framework runs on the robot operating system (ROS). Two coordinate frames, *base_link* and *odom*, reference the vehicle’s position in the environment. The Frenet reference frame is used to represent the positions of the traffic participants, thus enabling the trajectories and interactions to be described with fewer parameters.

### Lane Change Scenario Types

Our approach considers two lane change scenarios: cut-in and cut-out. A cut-in scenario is when a vehicle moves into the ego vehicle’s lane, while a cut-out scenario is when a front vehicle moves out of the ego vehicle’s lane. These scenarios are essential for assessing the performance of collision avoidance systems.

## 4. Edge Case Focused Concrete Scenario Generation

**Figure 1:**
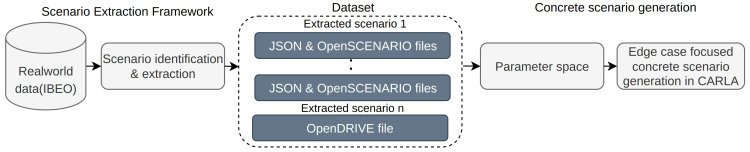
Architecture of the proposed method. The concrete scenario generation method is an end-to-end approach that involves identifying and converting real-world scenarios into a parametric representation to build a dataset and generate concrete scenarios from these parameters.

This section describes our methodology for generating concrete scenarios, which involves extracting lane change scenarios from real-world data and generating edge case concrete scenarios. The process, as shown in Figure 1, consists of two stages.

### 4.1. Lane Change Scenario Extraction

**Figure 2:**
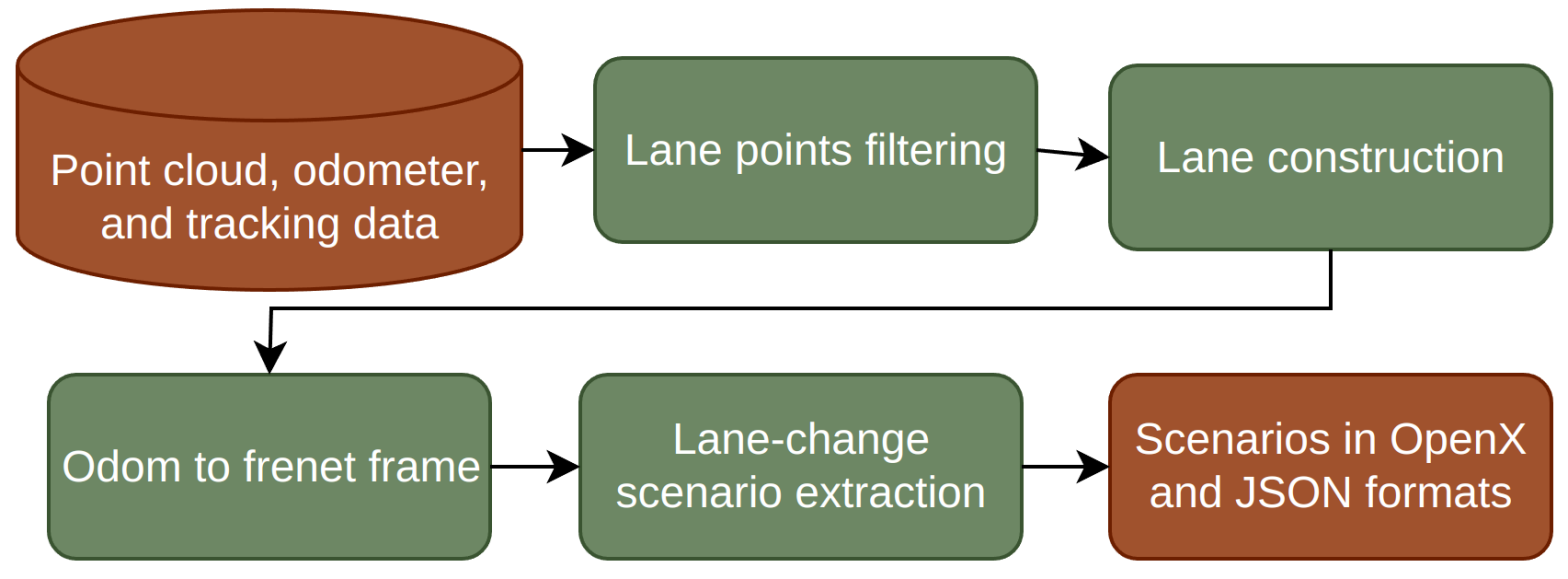
The scenario extraction approach pipeline involves the identification and parameterization of lane change scenarios in an open format using the data logged by the sensor system.

In the initial stage, we propose a novel approach for extracting lane change scenarios from real-world data and represent them in a parameterized form. The set of parameters, previously introduced in our work [13], characterizes the scenarios. We transform the extracted parameterized collections of lane change interactions into OpenSCENARIO files, thus allowing us to replay the trajectories in a simulator. The proposed scenario extraction technique utilizes the point clouds obtained from the rear, downwards-facing SICK lidar that are configured in a push-broom layout in combination with odometry and object tracking data. This module generates individual scenarios in the OpenSCENARIO format and their corresponding road structure in the OpenDRIVE format. The values of each parameter are stored in JSON files. The framework of the scenario extraction is depicted in Figure 2.

#### 4.1.1. Tracking and Point Cloud Processing

The data collection vehicle is equipped with an object-tracking system to capture the road participants’ trajectories in real-time. We used the *TF* transformation module in the robot operating system (ROS) to convert the tracking relative observations from the *base_link* (ego vehicle) frame to the *odom* (map) frame.

We used the lidar readings to find the location of the road lane markings, which helped us to find where and when a lane change occurs. To achieve this, we filtered the point cloud data from the push-broom lidar. Initially, the lidar points were sorted from the road center toward the lane boundaries. Then, we evaluated the first and second derivatives of the angle between the adjacent points to obtain the points hitting the road [32]. This allowed us to identify the curbs, as well as the separate road and non-road points. We grouped the points belonging to the lane markings by utilizing the intensity information in the point cloud. The reflective paint used to draw lane markings typically produces higher intensity readings than other road points.

After identifying and grouping the lidar points belonging to the lane markings, we converted them to the global *odom* frame and merged them to form the lanes. A more detailed explanation of this process can be found in [33]. We assigned a numerical label to each lane, and its location and tracking information were converted into a Frenet frame, where the data is represented in longitudinal *s* and lateral displacement *t*. This lane representation allows us to detect lane change scenarios based on specific criteria such as a lateral displacement to the lane center, which is not directly available in the Cartesian coordinate system.

#### 4.1.2. Scenario Extraction Logic and Parameters

To detect the lane change scenarios, we compared the lane number and lateral distance of the tracked vehicles to the ego path at each time step. A cut-in scenario was detected when the lateral displacement between any front and side vehicle and the ego vehicle lane approached zero, while a cut-out scenario was detected when a tracked vehicle moved away from the ego vehicle’s lane and the lateral displacement increased. The scenarios’ duration was set from eight seconds prior to the lane crossing to four seconds ahead, thereby capturing a total of 12 s of trajectory for each scenario, which is longer than the average lane change duration estimated by Toledo et al. [34].

To parameterize the real-world lane change scenarios, we used the list of parameters from our previous work [13]. These parameters came from four control points: *scenario start*, *cut start*, *cut end*, and *scenario end*. The scenario set the initial parameters for the ego and adversary vehicles, and the remaining parameters configured the adversary vehicle’s dynamic properties over time.

The output of this stage was the parametric representation of the extracted scenarios, which we used to create OpenSCENARIO files that described the trajectories in simulation. We also used OpenDRIVE files to describe the road network where the trajectories were executed. To replay the parameterized scenarios, we used the OpenSCENARIO player Esmini [35]. Additionally, we stored the representations of each captured scenario in the JSON files to facilitate the construction of the parameter space in the subsequent stage. The collection of OpenSCENARIO, OpenDRIVE, and JSON files have been made publicly available [33].

### 4.2. Edge Case Scenario Generation

**Figure 3:**
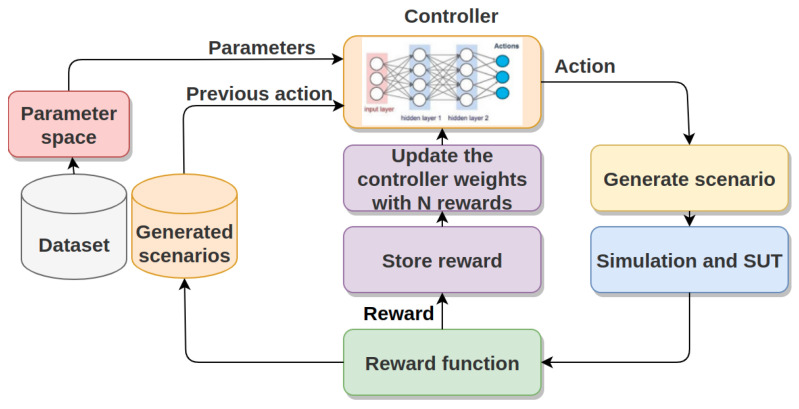
Architecture of the concrete scenario generation technique.

The proposed method for concrete scenario generation involves building a parameter search space based on the built scenario dataset and employing a reinforcement learning (RL)-based approach, as illustrated in Figure 3. Specifically, this work focused on cut-in scenarios, although the same process can be used for other road events.

The parameter space comprises the set of possible values for each parameter to generate new scenarios. In our previous work, independent distributions were introduced for each of the five parameters, and these were randomly sampled to learn the combination of the values that led to problematic scenarios. However, this approach was unable to account for parameter correlations, thus potentially resulting in unrealistic scenarios. To overcome this issue, this paper employed a multivariate–multimodal distribution to model the values of seven parameters that describe the extracted scenarios, thereby providing a more realistic range of values that account for the correlations observed in real-world vehicle interactions. The parameters used to recreate lane-changing vehicle trajectories were adversary vehicle trigger distance, velocity at the cut start, duration from start to cut start, velocity at the cut end, time from cut start to cut end, final velocity, and the duration from cut end to scenario end.

Algorithm 1 outlines the RL-based technique for generating concrete scenarios. In earlier stages, for each episode, the controller predicts parameter values as actions to generate a new concrete scenario in simulation by sampling from the parameter space. In later stages, the controller indicates actions based on the learned policy.

The state in the RL context encapsulates the current conditions of the environment. For the concrete scenario generation focused on lane changing, the state can be represented as a vector of the current values of the following seven parameters: adversary vehicle trigger distance, velocity at the cut start, duration from start to cut start, velocity at the cut end, time from cut start to cut end, final velocity, and the duration from cut end to scenario end. Formally, the state at time *t* can be represented as

(1) $$S_{t}=\left[d_{trigger},v_{start},t_{start},v_{end},t_{end},v_{final},t_{scenario}\right].$$

The action taken by the controller is to generate a new set of parameter values for the next scenario. Thus, an action $A_{t}$ at time *t* can be represented as a vector of the new parameter values:

(2) $$A_{t}=\left[d_{trigger}^{\prime },v_{start}^{\prime },t_{start}^{\prime },v_{end}^{\prime },t_{end}^{\prime },v_{final}^{\prime },t_{scenario}^{\prime }\right]$$

**Algorithm 1:** Pseudocode for the Proposed Concrete Scenario Generation Method
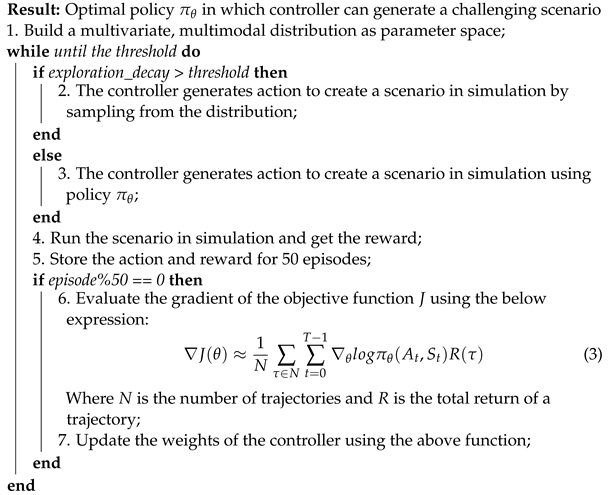


The reward function guides the learning process by considering the minimum safe distance using RSS and the occurrence of collisions. The reward function quantifies the risk in the lane change maneuver. A high reward signifies a dangerous lane change, while a low reward means the vehicles are driving safely. At the end of each episode, the total number of high-risk timesteps is normalized between −0.1 and 0.1, which is denoted as n_highrisk in the reward function.

(4) $$R=\{\begin{array}{cc} n\_highrisk & non\;collision\\ 0.25 & \;collision \end{array}$$

Through experimentation, we found that a smaller reward value improves exploration, resulting in a better chance of finding challenging scenarios, hence the values in the reward function. The controller is updated after storing action–reward pairs for 50 episodes, and the NN’s weights are updated to lead toward an optimal policy. The controller operates for 2500 episodes in this configuration.

The objective function J(θ) was used to evaluate and improve the policy. It was approximated using the total return *R* of trajectories over a batch of episodes and $log\pi_{\theta }$. The $log\pi_{\theta }$ in the objective function is equivalent to categorical cross entropy, so we can use categorical cross entropy as a loss function. The loss function is multiplied with return *R* to enable the learning in the direction of the maximum return. The gradient of *J* with respect to θ guides the update of the policy parameters, and it can be represented as follows:

(5) $$\nabla J\left(\theta \right)\approx \frac{1}{N}\sum_{\tau \in N}\sum_{t=0}^{T-1}\nabla_{\theta }log\pi_{\theta }\left(A_{t},S_{t}\right)R\left(\tau \right).$$

The controller (policy) iteratively samples actions (sets of parameter values) from the parameter space, and the environment provides feedback in the form of rewards based on the riskiness of the generated lane-changing scenarios. The goal is to learn an optimal policy that maximizes the likelihood of generating challenging and realistic concrete scenarios.

## 5. Results

The proposed method uses a data-driven parameter space constructed from the parametric representations of real-world events to generate problematic scenarios. This section presents the results of the scenario extraction process from the collected data and the RL learning of high-risk scenarios.

### 5.1. Scenario Parameterization

We developed a novel technique for extracting lane change scenarios from real-world data. The parameterized cut-in scenarios were replayed using the Esmini player. The positions of the ego and challenging vehicles were recorded at six timesteps during a cut-in maneuver.

**Figure 4:**
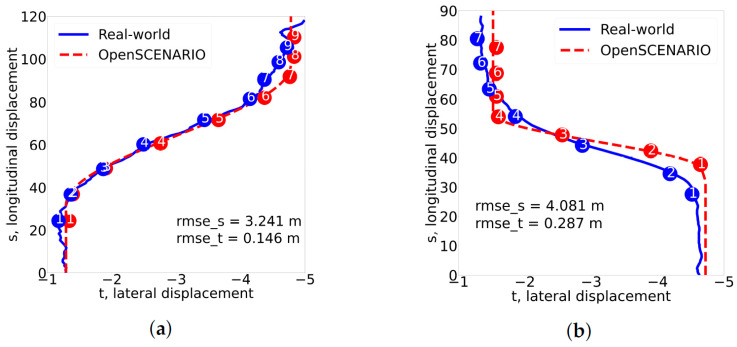
Alignment of the temporal (seconds from the cut start indicated within circles) and spatial parameters between the real-world scenarios and their corresponding parameterized cut-in and cut-out scenarios. *y* and *x* axis correspond to longitudinal and lateral displacement in meters. (**a**) Cut in. (**b**) Cut out.

To illustrate the comparison between the real-world scenario and its corresponding simulated version, Figure 4a shows the trajectories of a cut-in scenario. The blue trajectory represents the real-world data, and the red represents the parameterized scenario. The numbers within the circles indicate the seconds that have elapsed since the beginning of the lane change maneuver. The initial longitudinal positions of both trajectories were similar. Toward the end, at the 9th second, we can see a slight variation in the longitudinal position. Figure 4b shows a comparison of a parameterized cut-out scenario and its corresponding real-world scenario. Our scenario extraction method allowed us to build a dataset of lane change events from real-world data, which consists of 81 cut-in scenarios and 53 cut-out scenarios.

### 5.2. Training

**Figure 5:**
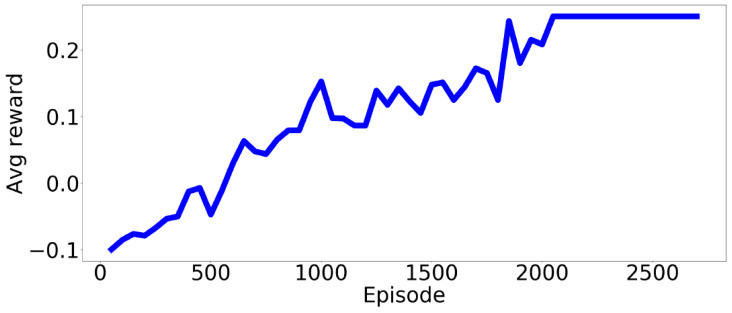
Average reward per episode showing the overall learning of the method.

The proposed method aims to maximize the reward function using RSS and a binary collision metric. The average reward was computed over 50 episodes. As shown in Figure 5, the reward tended to increase with additional iterations, thus indicating that the generated scenarios were of an increasingly higher risk. This suggests that the controller had learned to predict the action that generates a challenging scenario with maximum return, which, in this example, involved a collision.

**Figure 6:**
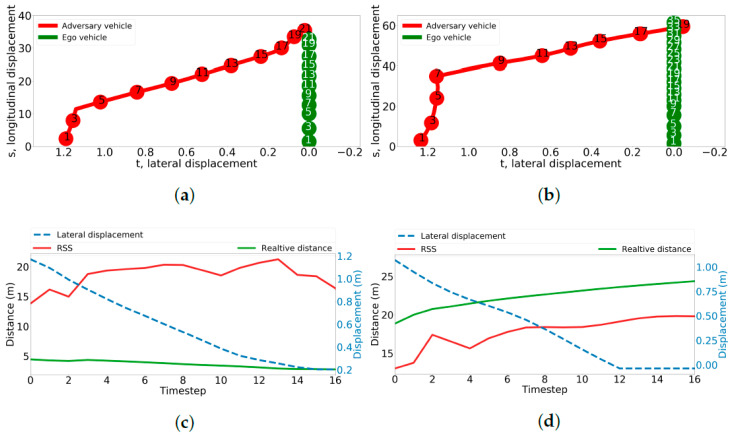
The method learned challenging and non-challenging cut-in scenarios from the multivariate, multimodal distribution, as illustrated in (**a**,**b**), where the *y* axis represents longitudinal displacement in meters, the *x* axis indicates lateral displacement in meters, and the numbers inside the circles denote the timesteps following the commencement of the cut-in. The safe threshold for RSS is also shown along with the actual relative distance. In cases where the RSS distance was lower than the actual distance, it indicated a dangerous scenario. (**a**) Challenging scenario representation. (**b**) Non-challenging scenario representation. (**c**) RSS performance in a challenging scenario. (**d**) RSS performance in a non-challenging scenario.

Figure 6a,c show the highest-risk scenario learned by the method. As shown in Figure 6a, the adversary vehicle initiated a lane change maneuver around the 4th timestep and ended up in a collision with the ego vehicle on the 21st. The minimum safe distance computed using longitudinal RSS is always higher than the actual relative distance between the vehicles, thus indicating a higher risk profile. The parameter values for this scenario are shown in Table 1. Upon analysis, we found that the collision occurred due to the restricted field of view of the SUT, which failed to detect and respond to the adversary vehicle in the blind spot.

In contrast, an example of a low-risk scenario is illustrated in Figure 6b,d. In Figure 6b, the adversary vehicle initiated a lane change maneuver at the 7th timestep and reached the ego vehicle’s lane around the 18th timestep. The comparison of both vehicles’ longitudinal positions at the 19th timestep indicated that the longitudinal position of the ego vehicle was safely behind the adversary vehicle. Figure 6d illustrates the risk profile of a non-challenging scenario, where the minimum safe distance computed by RSS is always lower than the relative distance between the ego vehicle and adversary vehicle, suggesting lower levels of risk.

**Figure 7:**
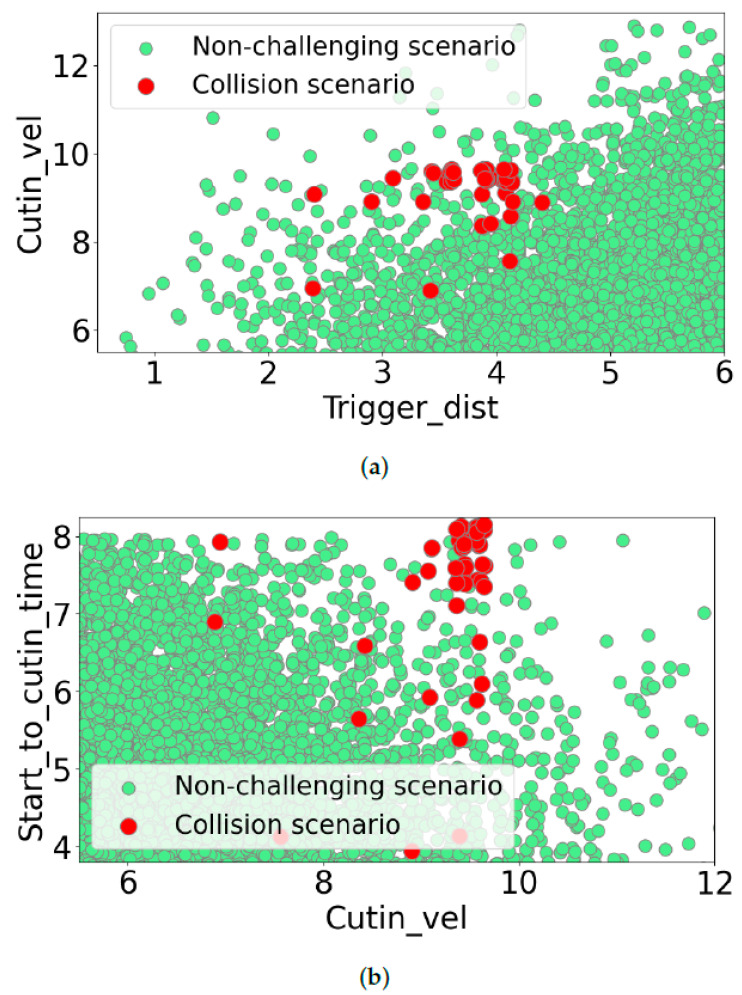

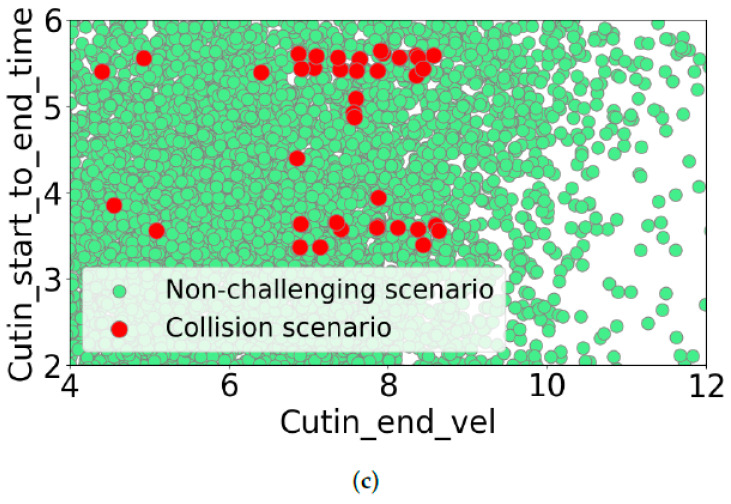
These graphs illustrate the multivariate, multi-modal distribution that represents the seven-dimensional parametric representation of the dataset. Green dots indicate normal scenarios, while red dots indicate collisions. The distribution is not uniform, and the correlations between the parameters are captured. The generated scenarios from this distribution are more realistic than those from uniform random sampling as they are more similar to the captured dataset. The parameter pairs shown in the graphs highlight the different correlations in the dataset. (**a**) Cutin_vel vs. trigger_dist; (**b**) start_to_cutin_time vs cutin_vel; and (**c**) cutin_start_to_end vs. cutin_end_vel.

As the seven parameters defined each scenario, the correlation between these parameters was encoded in a seven-dimensional space, which cannot be visualized directly. To show a subset of this distribution, we used two-dimensional plots, as illustrated in Figure 7. The green dots indicate the samples from the distribution that represent different collected scenarios, while the red dot shows the collision scenarios generated during the learning phase. The subfigures of Figure 7 illustrate the generated challenging scenarios and the distribution samples related to the following three pairs of scenario parameters: cutin_vel over trigger_dist (Figure 7a), start_to_cutin_time over cutin_vel (Figure 7b), and cutin_start_to_end_time over cutin_end_vel (Figure 7c). Our proposed method utilizes correlated data during the learning phase, thus leading to generated challenging scenarios that are consistent with the parameter space. In contrast, random sampling without considering the correlation can lead to unrealistic scenarios that are unlikely to occur in the real world.

### 5.3. Experiments

We compared three parameter spaces using independent univariate, multivariate normal, and multivariate multimodal distributions. In the first experiment, we fitted an independent multimodal distribution to each parameter. We used kernel density estimation to fit a multivariate normal distribution for the second experiment, while the third experiment used a multimodal, multivariate distribution to build the parameter space and to sample new high-risk scenarios.

**Figure 8:**
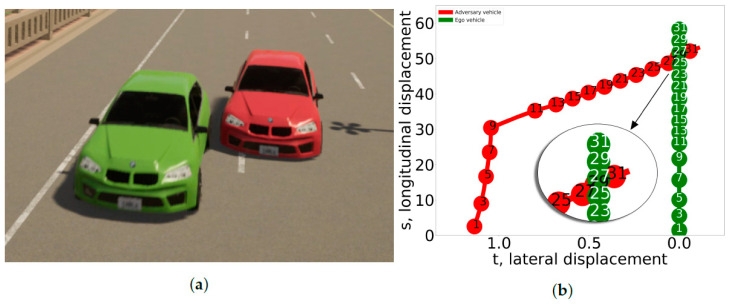

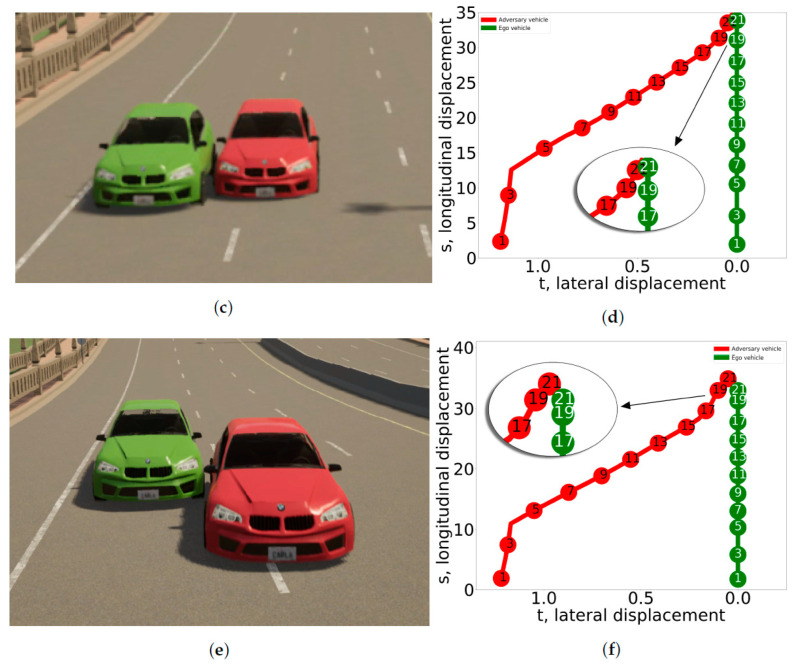
A qualitative analysis was conducted on the three different parameter spaces constructed using different representations of the dataset. The RL process output was used for each parameter space and a comparison was made. Figures (**a**,**b**) represent the learned scenario based on the independent univariate distribution, (**c**,**d**) represent the multivariate normal distribution, and (**e**,**f**) represent the multivariate multimodal distribution. The third parameter space, which is multivariate and multimodal, generated a collision interaction that closely matched the trajectory of the real-world data. The *y* axis represents longitudinal displacement in meters and the *x* axis represents lateral displacement in meters, with the numbers inside the circles indicating the timesteps following the commencement of the maneuver. (**a**) Independent. (**b**) Trajectory. (**c**) Multivariate. (**d**) Trajectory. (**e**) Multimodal. (**f**) Trajectory.

For the first experiment, we created a scenario by combining random samples from each univariate distribution. The resulting learned high-risk scenario, as shown in Figure 8a,b, depicted an adversary vehicle initiating a lane change maneuver when it was ahead of the ego vehicle but ending up in a collision by hitting the back of the ego vehicle. However, this type of interaction was not close to any of the scenarios from the real-world dataset, and it was not representative of a common scenario.

For the second experiment, we used kernel density estimation (KDE) to fit a multivariate normal distribution that incorporated the correlations between the different variables. The resulting collision scenario, as shown in Figure 8c,d, was closer to the trajectories collected in the real-world dataset. However, the vehicle collided with the side of the ego vehicle, and the adversary vehicle was slightly behind the ego vehicle at the 21st timestep, thereby resulting in a side collision. The sampling from the multivariate, unimodal normal distribution to the data was not likely to accurately represent the original data as most of the parameters were multimodal.

For the third and final experiment, we used a multimodal, multivariate distribution to describe the parameters in the dataset. We created these distributions to build the parameter space and sampled from them to generate and extract new high-risk scenarios. The resulting trajectory from our RL-based algorithm, as shown in Figure 8e, was much closer to the original real-world dataset. The learned parameters were close to the original data, and the algorithm was able to find a collision that was a cut-in scenario where the adversary vehicle misjudged the merge and was slightly too close to the ego vehicle. Figure 8f shows that the adversary vehicle began the lane change maneuver around the 4th timestep and collided with the ego vehicle around the 21st.

We conducted a quantitative analysis to compare the three different parameter spaces based on their likelihood estimates. We employed the RL-based algorithm to generate high-risk scenarios and used the Python library scipy to compute the likelihoods. We compared the likelihoods of the scenarios generated using independent univariate, multivariate normal and multivariate multimodal distributions. We found that the multivariate, multimodal distribution had the highest likelihood value of existing within the real-world data, which we believe better represents the collected data. Therefore, we used it as the ground truth model to compare the likelihood of a learned high-risk scenario. We fitted the distributions using the gaussian_kde function, and we computed the likelihood of the selected scenarios in the distribution using the pdf function. The likelihood for the converged RL scenario using the independent univariate distribution was extremely low ($e^{-95}$). This result indicated that the learned scenarios based on this approach significantly deviated from the original real-world dataset. The experiment based on the multivariate normal parameter space gave a significantly higher likelihood of existing within the real-world data ($e^{-17}$). However, the likelihood was still far lower than the multivariate, multimodal distribution, which had the highest likelihood value ($e^{-12}$), thus indicating that the scenarios generated using this distribution were more realistic and representative of the original dataset.

## 6. Conclusions and Future Work

We propose a method that generates realistic and challenging scenarios in simulation by using a data-driven parameter space and RL-based technique. Parameterized scenarios from real-world data enable the reproduction of events and the building of data-driven parameter spaces that encode realistic traffic behaviors.

We compared the following three parameter spaces for generating high-risk scenarios in autonomous vehicle testing: independent univariate, multivariate normal, and multivariate multimodal distributions. The first experiment, using univariate distributions, produced unrealistic scenarios that did not match real-world data. The second experiment, employing a multivariate normal distribution, yielded more realistic side collision scenarios but still lacked accuracy in representing complex real-world situations. The third experiment was most successful using a multimodal, multivariate distribution, which closely mirrored real-world driving scenarios (particularly in simulating realistic lane change maneuvers).

Our quantitative analysis reinforced these findings, with the multimodal, multivariate distribution showing the highest likelihood of resembling real-world scenarios. This contrasted with the low likelihood values of the scenarios generated from univariate distributions. The multivariate normal distribution was better but less effective than the multimodal approach. Our research demonstrates that a multivariate, multimodal distribution is the most effective in creating realistic and challenging scenarios for autonomous vehicle testing, which is crucial for ensuring the safety and reliability of these systems in real-world conditions.

In future work, we plan to integrate more complex traffic situations to enhance the robustness of our models. We also see potential in developing a more interactive simulation environment, where the autonomous vehicle’s responses can dynamically alter the scenario in real-timedh, thus providing a more comprehensive assessment of its decision-making capabilities.

## Figures and Tables

**Table 1:** Parameter values for a challenging scenario.

Parameter	Challenging
*trigger_dist*	4.0 m
*cutin_vel*	9.5 m/s
*start_to_cutin_dist*	8.0 m
*cutin_end_vel*	7.5 m/s
*cutin_start_to_cutin_end_time*	5.5 s
*final_vel*	8.0 m/s
*cutin_end_to_final_time*	3.0 s

## Data Availability

The code, dataset, and videos are available online at https://github.com/dkarunakaran/concrete_scenario_generation_real_world.

## References

[1] Standard, SAE. J3016-Taxonomy and Definitions for Terms Related to Driving Automation Systems for on-Road Motor Vehicles. https://www.sae.org/standards/content/j3016_202104/preview/.

[2] de Gelder E., Paardekooper J.P. Assessment of automated driving systems using real-life scenarios. Proceedings of the 2017 IEEE Intelligent Vehicles Symposium (IV).

[3] Elrofai H., Worm D., den Camp O.O. (2016). Scenario identification for validation of automated driving functions. Advanced Microsystems for Automotive Applications.

[4] Ponn T., Breitfuß M., Yu X., Diermeyer F. Identification of Challenging Highway-Scenarios for the Safety Validation of Automated Vehicles Based on Real Driving Data. Proceedings of the 2020 Fifteenth International Conference on Ecological Vehicles and Renewable Energies (EVER), Monte-Carlo.

[5] Menzel T., Bagschik G., Maurer M. Scenarios for development, test and validation of automated vehicles. Proceedings of the 2018 IEEE Intelligent Vehicles Symposium (IV).

[6] Koopman P., Kane A., Black J. Credible autonomy safety argumentation. Proceedings of the 27th Safety-Critical Systems Symposium.

[7] Gangopadhyay B., Khastgir S., Dey S., Dasgupta P., Montana G., Jennings P. Identification of test cases for automated driving systems using Bayesian optimization. Proceedings of the 2019 IEEE Intelligent Transportation Systems Conference (ITSC).

[8] Althoff M., Lutz S. Automatic generation of safety-critical test scenarios for collision avoidance of road vehicles. Proceedings of the 2018 IEEE Intelligent Vehicles Symposium (IV).

[9] Koren M., Alsaif S., Lee R., Kochenderfer M.J. Adaptive stress testing for autonomous vehicles. Proceedings of the 2018 IEEE Intelligent Vehicles Symposium (IV).

[10] Barbier M., Renzaglia A., Quilbeuf J., Rummelhard L., Paigwar A., Laugier C., Legay A., Ibañez-Guzmán J., Simonin O. Validation of Perception and Decision-Making Systems for Autonomous Driving via Statistical Model Checking. Proceedings of the 2019 IEEE Intelligent Vehicles Symposium (IV).

[11] Zhao D., Huang X., Peng H., Lam H., LeBlanc D.J. (2017). Accelerated evaluation of automated vehicles in car-following maneuvers. IEEE Trans. Intell. Transp. Syst..

[12] Dosovitskiy A., Ros G., Codevilla F., Lopez A., Koltun V. CARLA: An Open Urban Driving Simulator. Proceedings of the 1st Annual Conference on Robot Learning.

[13] Karunakaran D., Berrio J.S., Worrall S., Nebot E. Parameterisation of lane-change scenarios from real-world data. Proceedings of the 2022 IEEE 25th International Conference on Intelligent Transportation Systems (ITSC).

[14] Karunakaran D., Berrio J.S., Worrall S., Nebot E.M. (2022). Critical concrete scenario generation using scenario-based falsification. arXiv.

[15] De Gelder E., Manders J., Grappiolo C., Paardekooper J.P., Den Camp O.O., De Schutter B. Real-world scenario mining for the assessment of automated vehicles. Proceedings of the 2020 IEEE 23rd International Conference on Intelligent Transportation Systems (ITSC).

[16] Krajewski R., Bock J., Kloeker L., Eckstein L. The highd dataset: A drone dataset of naturalistic vehicle trajectories on german highways for validation of highly automated driving systems. Proceedings of the 2018 21st International Conference on Intelligent Transportation Systems (ITSC).

[17] Xinxin Z., Fei L., Xiangbin W. CSG: Critical Scenario Generation from Real Traffic Accidents. Proceedings of the 2020 IEEE Intelligent Vehicles Symposium (IV).

[18] Tenbrock A., König A., Keutgens T., Weber H. The ConScenD Dataset: Concrete Scenarios from the highD Dataset According to ALKS Regulation UNECE R157 in OpenX. Proceedings of the 2021 IEEE Intelligent Vehicles Symposium Workshops (IV Workshops).

[19] Bezzina D., Sayer J. (2014). Safety Pilot Model Deployment: Test Conductor Team Report.

[20] Zhao D., Guo Y., Jia Y.J. Trafficnet: An open naturalistic driving scenario library. Proceedings of the 2017 IEEE 20th International Conference on Intelligent Transportation Systems (ITSC).

[21] Zhou R., Liu Y., Zhang K., Yang O. (2022). Genetic algorithm-based challenging scenarios generation for autonomous vehicle testing. IEEE J. Radio Freq. Identif..

[22] Liu Y., Zhang Q., Zhao D. A Reinforcement Learning Benchmark for Autonomous Driving in Intersection Scenarios. Proceedings of the 2021 IEEE Symposium Series on Computational Intelligence (SSCI).

[23] Lu C. Test Scenario Generation for Autonomous Driving Systems with Reinforcement Learning. Proceedings of the 2023 IEEE/ACM 45th International Conference on Software Engineering: Companion Proceedings (ICSE-Companion).

[24] de Gelder E., Hof J., Cator E., Paardekooper J.P., den Camp O.O., Ploeg J., De Schutter B. (2022). Scenario parameter generation method and scenario representativeness metric for scenario-based assessment of automated vehicles. IEEE Trans. Intell. Transp. Syst..

[25] Van Otterlo M., Wiering M. (2012). Reinforcement learning and markov decision processes. Reinforcement Learning.

[26] Levine S. Policy Gradients. In Proceedings of the CS 294-112: Deep Reinforcement Learning. http://rail.eecs.berkeley.edu/deeprlcourse-fa18/.

[27] Matlab (2023). Reinforcement Learning Using Deep Neural Networks. https://www.mathworks.com/help/deeplearning/ug/reinforcement-learning-using-deep-neural-networks.html#.

[28] Shalev-Shwartz S., Shammah S., Shashua A. (2017). On a Formal Model of Safe and Scalable Self-driving Cars. CoRR.

[29] OpenSCENARIO—Association for Standardization of Automation and Measuring Systems. https://www.asam.net/standards/detail/openscenario/.

[30] OpenDRIVE—Association for Standardization of Automation and Measuring Systems. https://www.asam.net/standards/detail/opendrive/.

[31] MSC Open Standards—Essential for Self-Driving. https://d10tcz9jtwksbg.cloudfront.net/wp-content/uploads/2020/02/Open-Standards-Essential-for-Self-Driving.pdf.

[32] Zhou W., Berrio J.S., Worrall S., Nebot E. (2020). Automated Evaluation of Semantic Segmentation Robustness for Autonomous Driving. IEEE Trans. Intell. Transp. Syst..

[33] Karunakaran D. Techniques to Generate Lanes and OpeSCENARIO & OpenDRIVE Files. https://github.com/dkarunakaran/concrete_scenario_generation_real_world/blob/master/README.md.

[34] Toledo T., Zohar D. (2007). Modeling duration of lane changes. Transp. Res. Rec..

[35] esmini OpenSCENARIO Player. https://github.com/esmini/esmini.

